# Maturity-Onset Diabetes of the Young Associated With a Pathogenic ABCC8 Variant: Expanding the Phenotypic Spectrum

**DOI:** 10.1016/j.aed.2025.04.001

**Published:** 2025-04-16

**Authors:** Milca S. Velásquez-Hernandez, Liany F. Acosta-Paguada, Paola Sophia Bonilla Medina, Eduardo Smelin Perdomo Domínguez

**Affiliations:** 1 Faculty of Medicine, Catholic University of Honduras, San Pedro Sula, Honduras; 2 Department of Pediatric Endocrinology, Hospital Bendaña, San Pedro Sula, Honduras; 3 Department of Pediatric Endocrinology, Hospital Mario Catarino Rivas, San Pedro Sula, Honduras; 4 Grupo de Investigación Médica de la Universidad Católica de Honduras, Faculty of Medicine, Catholic University of Honduras, San Pedro Sula, Honduras

**Keywords:** maturity-onset diabetes of the young, *ABCC8* mutation, monogenic diabetes, β-cell dysfunction, congenital hyperinsulinism

## Abstract

**Background/Objective:**

Monogenic diabetes results from single-gene sequence variants affecting β-cell function. While *ABCC8* sequence variants are linked to congenital hyperinsulinism (CHI) and neonatal diabetes, their role in maturity-onset diabetes of the young (MODY) is less defined. The objective of this report is to describe a patient with MODY caused by a heterozygous *ABCC8* c.4613G>A (p.Arg1538Gln) sequence variant, highlighting its phenotypic variability and implications for diagnosis and management.

**Case Report:**

A 9-year-old boy presented with progressive weight gain and obesity (body mass index 25.25 kg/m^2^, +2.32 SD). Fasting glucose was 94 mg/dL (reference: 70-100 mg/dL). Lifestyle modifications were recommended, but follow-up was not conducted.

At age 11, he returned with fatigue and daytime sleepiness. Laboratory results showed a fasting glucose level of 343 mg/dL, a β-hydroxybutyrate level of 1.0 mmol/L (reference: <0.6 mmol/L), and a hemoglobin A1C level of 8% (64 mmol/mol) (reference: <5.7% [<39 mmol/mol]). Diabetes autoantibodies were negative. C-peptide was 0.53 ng/mL (reference: 0.93-3.73 ng/mL), and postprandial insulin was 100.1 μIU/mL (reference: <60 μIU/mL). Insulin therapy was initiated.

Genetic testing confirmed a pathogenic *ABCC8* c.4613G>A (p.Arg1538Gln) variant. At follow-up, hemoglobin A1C improved to 6% (42 mmol/mol), and fasting glucose was 136.5 mg/dL.

**Discussion:**

*ABCC8* sequence variants exhibit a broad phenotypic spectrum, ranging from CHI to MODY. While the c.4613G>A (p.Arg1538Gln) variant has been previously associated with CHI, this case presents with MODY, highlighting its phenotypic variability.

**Conclusion:**

This case expands the phenotypic spectrum of *ABCC8*-related diabetes, demonstrating that the c.4613G>A (p.Arg1538Gln) variant can present as MODY without prior CHI. Genetic testing is essential for accurate diagnosis and treatment strategies in monogenic diabetes.

**Highlights**

• ABCC8 sequence variants exhibit a broad phenotypic spectrum, ranging from congenital hyperinsulinism to maturity-onset diabetes of the young
• First-degree relatives may be unaffected despite familial diabetes history, suggesting variable expressivity or germline mosaicism
• Sulfonylurea efficacy in ABCC8-related diabetes depends on the specific sequence variant, with some variants responding effectively while others requiring alternative treatments

**Clinical Relevance**

An 11-year-old boy with an ABCC8 c.4613G>A (p.Arg1538Gln) sequence variant presented with maturity-onset diabetes of the young without prior neonatal hypoglycemia or hyperinsulinism. This case highlights the phenotypic variability of ABCC8 mutations and the importance of considering monogenic diabetes in pediatric patients with atypical diabetes presentations.

## Introduction

Monogenic diabetes is a heterogeneous group of disorders caused by single-gene sequence variants affecting pancreatic β-cell function and insulin secretion.^1^ It includes neonatal diabetes, maturity-onset diabetes of the young (MODY), and diabetes-associated syndromes. MODY is characterized by early-onset, nonautoimmune diabetes, typically diagnosed before 25 years of age, with strong family history and preserved β-cell function at diagnosis. Although MODY accounts for 0.5-5% of nonautoimmune diabetes, it is frequently underdiagnosed due to overlap with other types and limited genetic testing.^2^

The *ABCC8* gene (chromosome 11p15.1) encodes the sulfonylurea receptor 1, which regulates the ATP-sensitive potassium (KATP) channel in pancreatic β-cells.^3^ Sequence variants in *ABCC8* can lead to conditions ranging from congenital hyperinsulinism (CHI) to various diabetes forms, depending on their impact on KATP function.^4^ The p.Arg1538Gln variant, located in the nucleotide-binding domain 2 (NBD2) of sulfonylurea receptor 1, has been associated with CHI, but its link to MODY remains unclear.^3^

We report a case of *ABCC8*-related MODY due to a heterozygous p.Arg1538Gln (R1538Q) sequence variant, highlighting the phenotypic variability of *ABCC8*-related diabetes.

## Case Report

We report the case of a Honduran 11-year-old boy born to nonconsanguineous parents. He is the only child of a 40-year-old mother and a 37-year-old father. The pregnancy was uncomplicated, and there was no known exposure to teratogenic drugs, infections, or radiation. He was delivered via cesarean delivery due to the presence of a nuchal cord, with a birth weight of 3.8 kg. His neonatal and early developmental milestones were unremarkable.

During infancy, he was diagnosed with a cow’s milk protein allergy, which resolved by age 9. At that time, he was also evaluated for severe urticaria, although allergy testing remained pending. At age 5, he underwent uneventful chalazion surgery. He was later diagnosed with attention-deficit/hyperactivity disorder and is currently managed with occupational therapy. Family history is significant for type 2 diabetes in second-degree relatives (maternal and paternal grandparents), although neither parent is diabetic.

When he was 9 years old, the patient presented for evaluation due to obesity and metabolic alterations. Anthropometric measurements revealed a weight of 44 kg (92nd percentile, +1.43 SD), a height of 132 cm (31st percentile, −0.51 SD), and a body mass index (BMI) of 25.25 kg/m^2^ (98th percentile, +2.32 SD). Body composition showed a fat mass of 15.3 kg (34.8% of total weight), exceeding the ideal by 9.5 kg. Additional findings included an abdominal circumference of 80 cm and triceps skinfold thickness of 20 mm, both above the 99th percentile. Physical examination revealed acanthosis nigricans on the posterior neck and Tanner stage 1/1.

Laboratory tests showed a fasting glucose level of 94 mg/dL (reference: 70-100 mg/dL), vitamin D insufficiency (22.9 ng/mL, optimal: >30 ng/mL), and a lipid profile revealing low-density lipoprotein 74 mg/dL (reference: <100 mg/dL), high-density lipoprotein 38 mg/dL (reference: ≥40 mg/dL), triglycerides 75 mg/dL (reference: <150 mg/dL), and total cholesterol 128 mg/dL (reference: <170 mg/dL).

Initial management included dietary sugar restrictions, vitamin D supplementation (400 IU/d), and laboratory monitoring of glucose, insulin, hemoglobin A1C (HbA1C), and liver function tests. However, no follow-up evaluations were conducted.

Two years later, the patient returned due to fatigue and daytime sleepiness. Laboratory results revealed hyperglycemia (fasting glucose: 343 mg/dL), mild ketonemia (β-hydroxybutyrate: 1.0 mmol/L, normal <0.6 mmol/L), and elevated HbA1C (8% [64 mmol/mol], reference: <5.7% [<39 mmol/mol]). Anthropometric measurements showed a weight of 51.5 kg (89th percentile, +1.26 SD), a height of 140.5 cm (32nd percentile, −0.49 SD), and a BMI of 26.09 kg/m^2^ (97th percentile, +2.01 SD). A diagnosis of diabetes was established. Initial management included subcutaneous insulin (Tresiba 22 units daily) and fluid resuscitation.

Further testing included diabetes autoantibodies (glutamate decarboxylase, insulin autoantibodies, IA-2, and islet cell antibodies), C-peptide, and basal/postprandial insulin levels.

Results revealed negative autoantibodies, low C-peptide (0.53 ng/mL, normal: 0.93-3.73 ng/mL), elevated fasting insulin (88.7 μIU/mL, normal: 2.60-24.90 μIU/mL), and postprandial hyperinsulinemia (100.1 μIU/mL, normal: <60 μIU/mL). At the time of testing, the patient was receiving long-acting basal insulin. Insulin was adjusted to 12 units daily following recurrent episodes of post-exercise hypoglycemia. A MODY genetic panel was recommended for further evaluation.

At follow-up, anthropometric measurements were weight 52 kg (88th percentile, +1.22 SD), height 141 cm (29th percentile, −0.57 SD), and BMI 26.16 kg/m^2^ (97th percentile, +1.98 SD). Laboratory tests showed a fasting glucose level of 102 mg/dL and an HbA1C level of 7% (53 mmol/mol). Liver function tests revealed alanine transaminase 324.7 U/L (reference: <40 U/L) and aspartate transaminase 174.8 U/L (reference: <35 U/L). Vitamin E and omega-3 supplementations were initiated for suspected hepatic dysfunction.

**Fig. 1:**
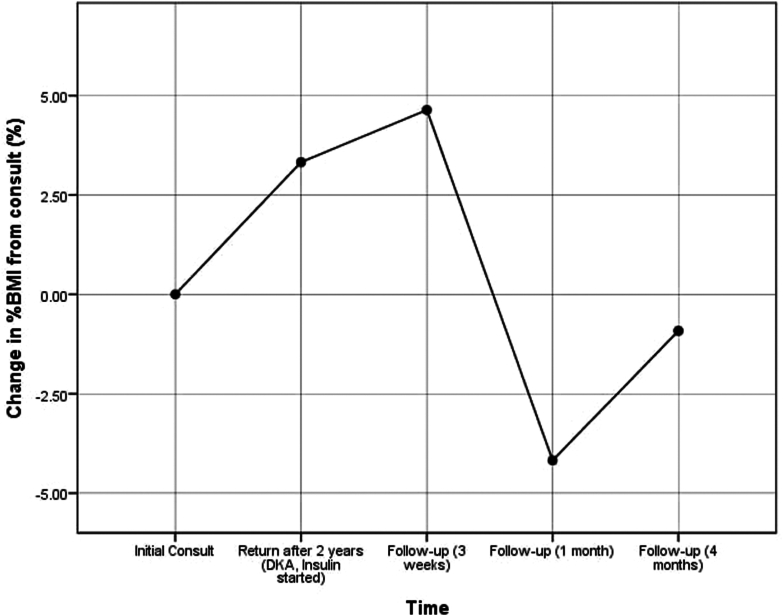
Change in body mass index (BMI) (%) relative to the initial consultation, highlighting weight management trends over time. *DKA* = diabetic ketoacidosis.

**Fig. 2:**
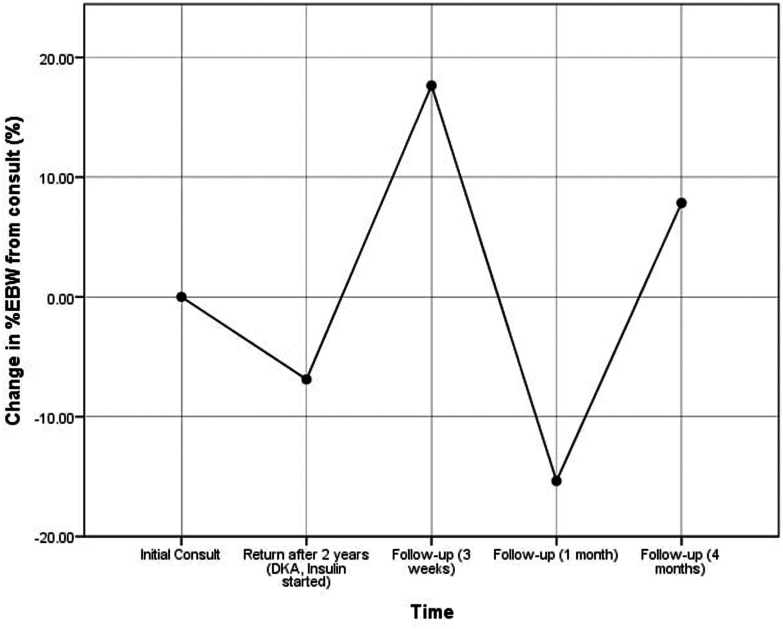
Change in excess body weight (%EBW) relative to initial consultation, showing variability in body composition adjustments. *DKA* = diabetic ketoacidosis.

Genetic testing identified a pathogenic heterozygous *ABCC8* c.4613G>A (p.Arg1538Gln) variant and a *GATA6* c.868G>T (p.Ala290Ser) variant of uncertain significance. At that time, HbA1C improved to 6% (42 mmol/mol), with a fasting glucose level of 136.5 mg/dL. Liver function tests showed an aspartate transaminase level of 34.9 U/L and an alanine transaminase level of 39.7 U/L. Anthropometric measurements showed a weight of 53 kg (87th percentile, +1.16 SD), a height of 143 cm (31st percentile, −0.52 SD), and a BMI of 25.92 kg/m^2^ (96th percentile, +1.86 SD). BMI and excess weight changes over time are shown in Figures 1 and 2. The follow-up plan included continuous glucose monitoring, lipid profile evaluations, and repeat HbA1C testing.

## Discussion

This case highlights a pathogenic *ABCC8* variant, c.4613G>A (p.Arg1538Gln), located in the NBD2 domain, associated with MODY. While *ABCC8* sequence variants have been extensively studied in neonatal diabetes and CHI, their role in MODY remains less well-established. Our patient presented with MODY without prior neonatal hypoglycemia or hyperinsulinism, which contrasts with typical presentations associated with this variant.

The c.4613G>A (p.Arg1538Gln) variant has been described as a heterozygous, dominant, loss-of-function sequence variant associated with nonneonatal diabetes mellitus. Among reported cases, 39% of individuals with *ABCC8-*nonneonatal diabetes mellitus were diagnosed between the ages of 6 and 18, consistent with our patient’s diagnosis at age 11. Although not statistically significant (*P* = .960), the mean diagnosis age for inactivating variants (19.1 ± 10.0 years) was lower than that for activating ones (28.9 ± 11.6 years), which further supports our case.^5^

Although MODY is classically associated with normal or mildly increased weight, obesity can coexist with monogenic diabetes. A study found that 4.5% of overweight or obese adolescents initially diagnosed with type 2 diabetes were subsequently found to have MODY.^6^ These findings show that MODY can occur alongside insulin resistance and metabolic syndrome features, especially in genetically predisposed individuals.

Sequence variants in NBD2 affect ATP-mediated inhibition of the KATP channel, disrupting insulin secretion. The c.4613G>A (p.Arg1538Gln) variant has been previously reported in CHI, often associated with macrosomia and early-onset hyperinsulinism.^7^^,^^8^ However, our patient had a normal birth weight and no neonatal hypoglycemia. A systematic review of *ABCC8* sequence variants found that 32% of probands had a birth weight <2,500 g, while only 1% exceeded 4,000 g, further emphasizing the phenotypic variability observed in this condition.^5^

A strong family history of diabetes in second-degree relatives supports the notion of progressive β-cell dysfunction in this case. It is possible that undiagnosed mild hypoglycemia in previous generations preceded later-onset diabetes, as seen in our patient.^4^^,^^9^ Similar biphasic progressions—early mild hypoglycemia followed by diabetes—have been described in *ABCC8-*related disorders.^3^^,^^10^^,^^11^ Moreover, dominant *ABCC8* sequence variants demonstrate significant phenotypic variability within and across families, with some individuals developing neonatal hyperinsulinism and others diabetes later in life.^4^^,^^12^ Table^7^^,^^8^^,^^11^ summarizes the phenotypic characteristics of previously reported cases with the same *ABCC8* c.4613G>A (p.Arg1538Gln) variant, alongside our patient.

**Table:** Cases with the c.4613G>A (p.Arg1538Gln) variant in ABCC8

Patient	Age of presentation	Birth weight	Clinical phenotype	Treatment
This case report	11 y	3.8 kg	MODY	Insulin
Wang et al^8^	Neonatal period	4.8 kg	CHI	Diazoxide
Kapoor et al^11^	47 y	Not reported	Type 2 diabetes	Metformin + glicazide
Kapoor et al^11^	39 y	NA	Type 2 diabetes	Metformin
Kapoor et al^11^	Neonatal period	NA	CHI	Diazoxide
Kapoor et al^11^	Neonatal period	NA	Transient neonatal hypoglycemia	Not required
Park et al^7^	Neonatal period	Not reported	CHI	Diazoxide

Abbreviations: CHI = congenital hyperinsulinism; MODY = maturity-onset diabetes of the young; NA = not applicable.

Reported cases of individuals with the ABCC8 c.4613G>A (p.Arg1538Gln) mutation, including the age of onset, clinical phenotype, and treatment approaches.

*ABCC8* sequence variants are typically autosomal dominant, but nearly 50% arise *de novo*.^13^ Sequence variants in ABCC8, including *de novo* variants such as Q485R, have been reported in patients without affected first-degree relatives.^14^ This highlights that a positive family history is not essential for the diagnosis of monogenic diabetes, reinforcing the importance of genetic testing even in the absence of a classic inheritance pattern. In our case, genetic testing of the parents was not performed, but neither had diabetes, while both sets of grandparents were affected. This raises the possibility of silent transmission through germline mosaicism or reduced penetrance, as described in *ABCC8*- and *KCNJ11*-related monogenic diabetes.^15^

Sulfonylureas are effective in ∼73% of ABCC8-related diabetes cases, but response varies by sequence variants.^5^ While ∼90% of neonatal diabetes cases caused by KATP channel sequence variants have successfully transitioned from insulin to sulfonylureas, not all MODY-associated *ABCC8* variants respond adequately.^16^^,^^17^ For instance, the p.Arg797Gln variant has demonstrated poor responsiveness to sulfonylureas, likely due to its location within the ABC transporter domain.^18^ Given the absence of functional studies, insulin therapy was selected as the initial management strategy. Future investigations into sulfonylurea responsiveness in MODY cases with this variant could provide valuable insights for optimizing treatment.

Our patient also carried a *GATA6* c.868G>T (p.Ala290Ser) variant of uncertain significance. While *GATA6* sequence variants have been associated with pancreatic agenesis and neonatal diabetes, no structural pancreatic abnormalities were found.^19^ The potential interplay between *ABCC8* and *GATA6* sequence variants in β-cell dysfunction remains unclear, warranting further exploration to determine its clinical relevance.

This case expands the phenotypic spectrum of *ABCC8*-related diabetes, demonstrating that the c.4613G>A (p.Arg1538Gln) sequence variant can present as MODY without neonatal hypoglycemia or hyperinsulinism. Given the heterogeneous clinical presentation, genetic testing remains crucial for accurate diagnosis and treatment. Future research should explore the potential interplay between *ABCC8* and *GATA6* sequence variants in β-cell dysfunction.

## Disclosure

The authors have no conflicts of interest to disclose.
